# Increased expression of *pgph-1*, T23F2.4, and *cyp-14A5* in *C. elegans* *dpy-7* mutants and by high salt

**DOI:** 10.17912/micropub.biology.000136

**Published:** 2019-08-29

**Authors:** Gabrielle Scolaro, Kelsey Bridges, Shayla Curry, Stephanie Jacobson, Marissa LoPresti, Katina Pappas, Nicolas Ramirez, Lindsay Savigne, Sarah Sherman, Katherine Upshaw, Erin Walsh, Keith Choe

**Affiliations:** 1 Department of Biology and Genetics Institute, University of Florida, Gainesville, Florida 32611

**Figure 1.**
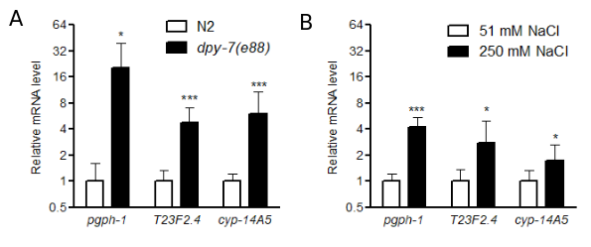
**Figure 1.** Relative mRNA expression levels of genes induced in *dpy-7(e88)* mutants (A) or by 250 mM NaCl (B). *P < 0.05 or ***P < 0.001, normalized by *rpl-2* and compared to the expression levels in N2 worms or worms exposed to 51 mM NaCl. N = 9 or 12 replicate pools of 10 or 20 worms.

## Description

Extracellular matrices (ECM) are ubiquitous features of metazoan tissues that have structural and signaling functions (Hay 1981). The cuticle of *C. elegans* is a complex and flexible ECM composed of over 100 cross-linked collagen fibers secreted by underlying epidermal cells. In addition to functioning as flexible exoskeletons, nematode cuticles act as barriers, providing the first line of defense against environmental stressors such as toxins, water imbalances, and pathogens (Page and Johnstone 2007).

It was previously reported that loss or mutation of some cuticle collagens caused constitutive activation of genes that are normally induced by environmental stressors such as high osmolarity and pathogens (Lamitina et al. 2006, Pujol et al. 2008, Choe 2013, Zugasti et al. 2016). We recently demonstrated that this effect is restricted to six collagens required for formation of circumferential bundles termed annular furrows and to activation of three conserved stress responses (Dodd et al. 2018). These results are consistent with an ECM damage sensor that is associated with annular furrows and signals to specific downstream environmental response pathways.

DPY-7 is one of the annular furrow collagens (Cox et al. 1980, McMahon et al. 2003, Thein et al. 2003). We recently used RNAseq analysis to identify candidate genes that may be activated by *dpy-7* mutation; these genes may contribute to stress responses when the cuticle is disrupted (Dodd et al. 2018). In the current study, we used quantitative RT-PCR to measure expression of stress response-related genes in *dpy-7(e88)* worms; mRNA levels of each potential stress response gene were normalized to *rpl-2*, which encodes a ribosomal protein*. * We selected genes based on predicted functions in stress responses and significant activation in *dpy-7* worms in prior RNAseq analyses (Dodd et al. 2018). Twelve biological replicates were measured for each strain, and each RNA replicate sample was isolated from either 10 N2 control or 20 *dpy-7* first day adult worms grown on agar seeded with OP50 bacteria. As shown in Figure 1A, *pgph-1*, T23F2.4, and *cyp-14A5* were significantly upregulated in *dpy-7* mutants compared to N2 in the absence of environmental stress. We also measured these mRNAs in worms exposed to high salt to determine if they are induced by a relevant environmental condition (Lamitina et al. 2006, Choe 2013, Dodd et al. 2018). As shown in Figure 1B, *pgph-1*, T23F2.4, and *cyp-14A5* were induced by 24 h exposure to 250 mM NaCl; nine biological replicates were measured for each condition.

*pgph-1* encodes an ortholog of glycerol-3-phosphate phosphatase (G3PP). G3PP was recently identified in mammalian cells and shown to catalyze the final step in synthesis of glycerol from products of glycolysis (Mugabo et al. 2016). In this role, G3PP functions at the nexus of glucose, fatty acid, redox, and ATP metabolism with expected roles in diabetes and obesity. *C. elegans* strongly increases synthesis of glycerol during high osmolarity stress, in part, by inducing *gpdh-1*; *gpdh-1* encodes an enzyme that functions upstream from G3PP by catalyzing production of glycerol-3-phosphate (Lamitina et al. 2004).

T23F2.4 encodes a close homolog of plant and fungal PMP3 (plasma membrane proteolipid 3), which are absent from most metazoans other than nematodes. Yeast and plant PMP3 homologs are induced by high salt, influence membrane potential, and are required for high salt resistance (Navarre and Goffeau 2000, Fu et al. 2012).

*cyp-14A5* encodes a cytochrome P450 family member that was previously shown to be required for resistance to pro-oxidants (Park et al. 2009). We recently demonstrated that other drug metabolism gene family members such as glutathione S-transferases and glucuronosyltransferases are also activated in furrow mutants (Dodd et al. 2018).

Our results expand the repertoire of osmotic and detoxification stress-related genes confirmed to be constitutively activated by mutation of cuticle annular furrow collagens or high osmolarity. Future studies will focus on *pgph-1* and T23F2.4. *pgph-1* is a model for regulation and function of G3PP, which is expected to play important roles in diverse human metabolic pathologies (Mugabo et al. 2016). Given that PMP3 homologs are absent from almost all metazoans except nematodes, T23F2.4 could be a rare case of horizontal gene transfer to a metazoan that confers environmental stress resistance. Stabilization of membrane potential by PMP3 proteins may have benefits for metazoan-specific excitable cells such as nerve and muscle.

## Methods

Evaluation and selection of stress-response genes, primer design, and measurement of mRNA after high salt exposure were conducted as part of an immersive five-week CURE course (course-based undergraduate research experience) named “Molecular and Genetic Responses to Environmental Stress” at the University of Florida (Auchincloss et al. 2014, Wang 2017). Primers were designed using Primer-BLAST (U.S. National Library of Medicine).

Wild type N2 and CB88 *dpy-7(e88)* mutant nematodes were grown as mixed populations at 20ºC on 51 mM NaCl NGM (nematode growth medium) agar plates with OP50 bacteria. First day gravid adult worms (10 per tube for N2 and 20 per tube for *dpy-7*) were picked into PCR tube lids containing buffer (51 mM NaCl, 0.2% Tween-20, 2.5 mM KH2PO4 pH 6.0), centrifuged into the bottom containing an equal volume of lysis buffer (10 mM Tris pH 8.0, 1.0% triton X-100, 1.0% Tween 20, 0.5 mM EDTA, 20 mg/ml proteinase K), frozen at -80ºC, and lysed at 65ºC for 10 min followed by 2 min at 80ºC to denature proteinase K. Promega GoScript Reverse Transcriptase (A5003) was used to synthesize cDNA with poly T primers according to manufacturer’s recommendations. qPCR was conducted with Biotium Forget-me-not™ qPCR master mix (31041) according to manufacturer’s recommendations. Relative mRNA levels were calculated using the ΔΔCT method adjusted with primer efficiencies calculated from standard curves. Statistical significance was determined with Student’s T-tests.

## Reagents

Strains:

*C. elegans* strains used were wild-type N2 Bristol and CB88 *dpy-7(e88)*; both are available at the CGC.

Primers:

T23F2.4 – GCTCTTCTTCTCCCGCCAG and CCGGGAATGTAGCCGAGAAT

*pgph-1* – TTGACGCTGATGGTGTCCTG and TGGTGGCATTATTGGTGAGCA

*cyp-14A5* – TTTGTAACGCAAGGTGACGC and CTCCTGTGTTTGGATGGGGT

*rpl-2* – CTTTCCGCGACCCATACAA and CACGATGTTTCCGATTTGGAT
